# Supplementary material for: The Pharmaceutical Ability of Pistacia lentiscus L. Leaves Essential Oil Against Periodontal Bacteria and Candida sp. and Its Anti-Inflammatory Potential
Source: Antibiotics (Basel). 2020 May 26;9(6):281. doi: 10.3390/antibiotics9060281 (PMC7345856; doi:10.3390/antibiotics9060281)
Supplement: Supplementary file 1 [file antibiotics-09-00281-s001.pdf]

The pharmaceutical ability of Pistacia lentiscus L. leaves essential oil against periodontal bacteria and Candida sp. and its anti-inflammatory potential

Egle Milia, Marianna Usai, Barbora Szotakova, Marie Elstnerova, Věra Králová, Guy D'hallewin, Ylenia Spissu, Antonio Barberis, Mauro Marchetti, Antonella Bortone, Vincenzo Campanella, Giorgio Mastandrea, Lenka Langhansova, Sigrun Eick

Supplementary Material

Cyclic voltammetries of the three most represented phenolics of *Pistacia lentiscus L.* essential oil

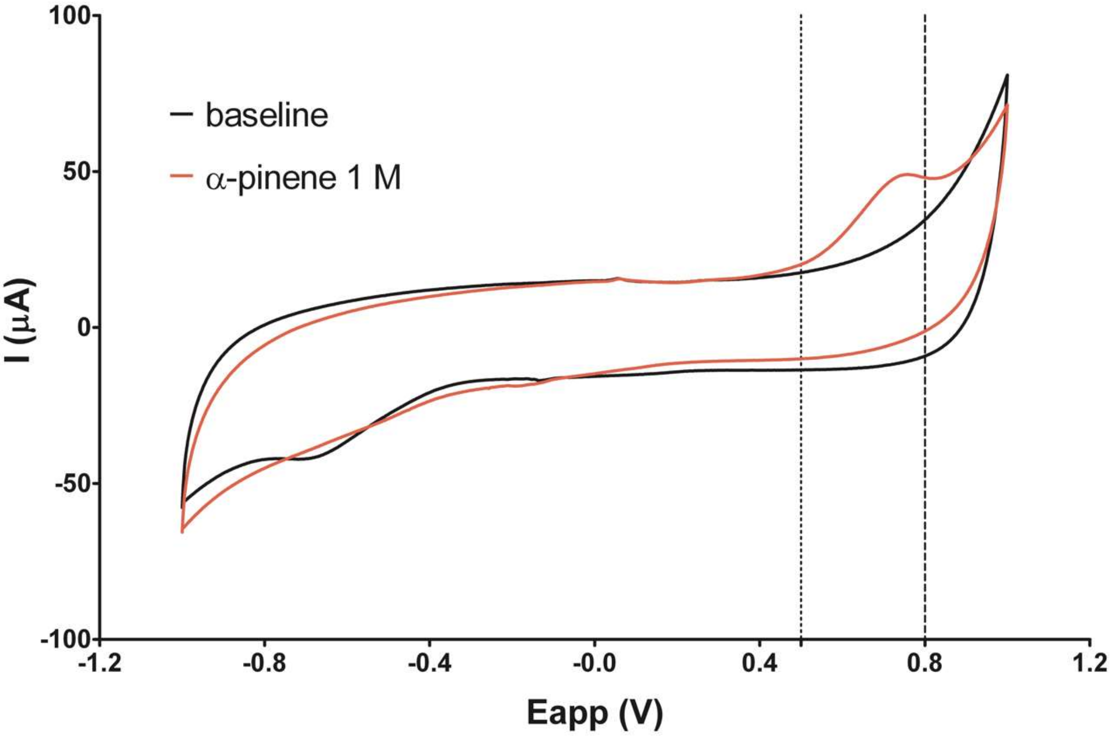

Figure S1. Cyclic voltammetry of α-pinene with a scanned potential range (E_{app}) comprised between - 1 V and + 1 V *vs* Ag/AgCl reference electrode, in the absence (black line) and in the presence of 1M α-pinene (red line).

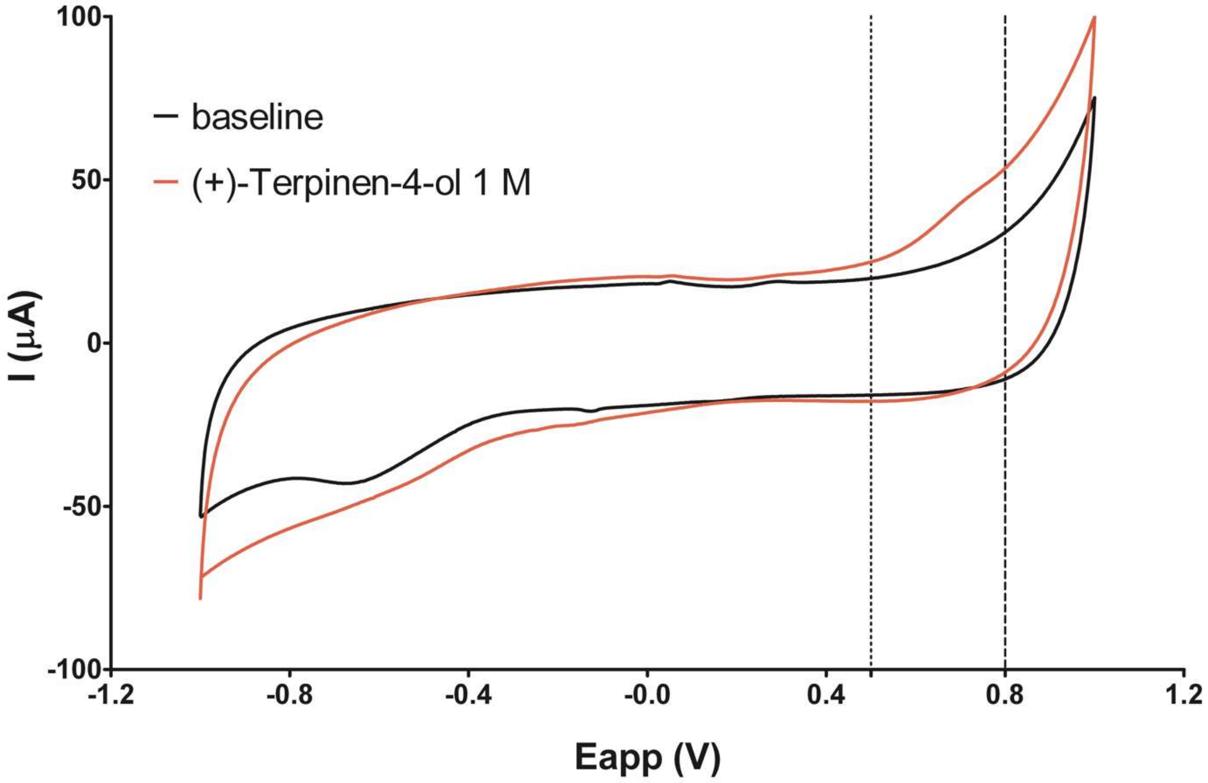

Figure S2. Cyclic voltammetry of terpinen-4-ol with a scanned potential range (E_{app}) comprised between - 1 V and + 1 V *vs* Ag/AgCl reference electrode, in the absence (black line) and in the presence of 1M terpinen-4-ol (red line).

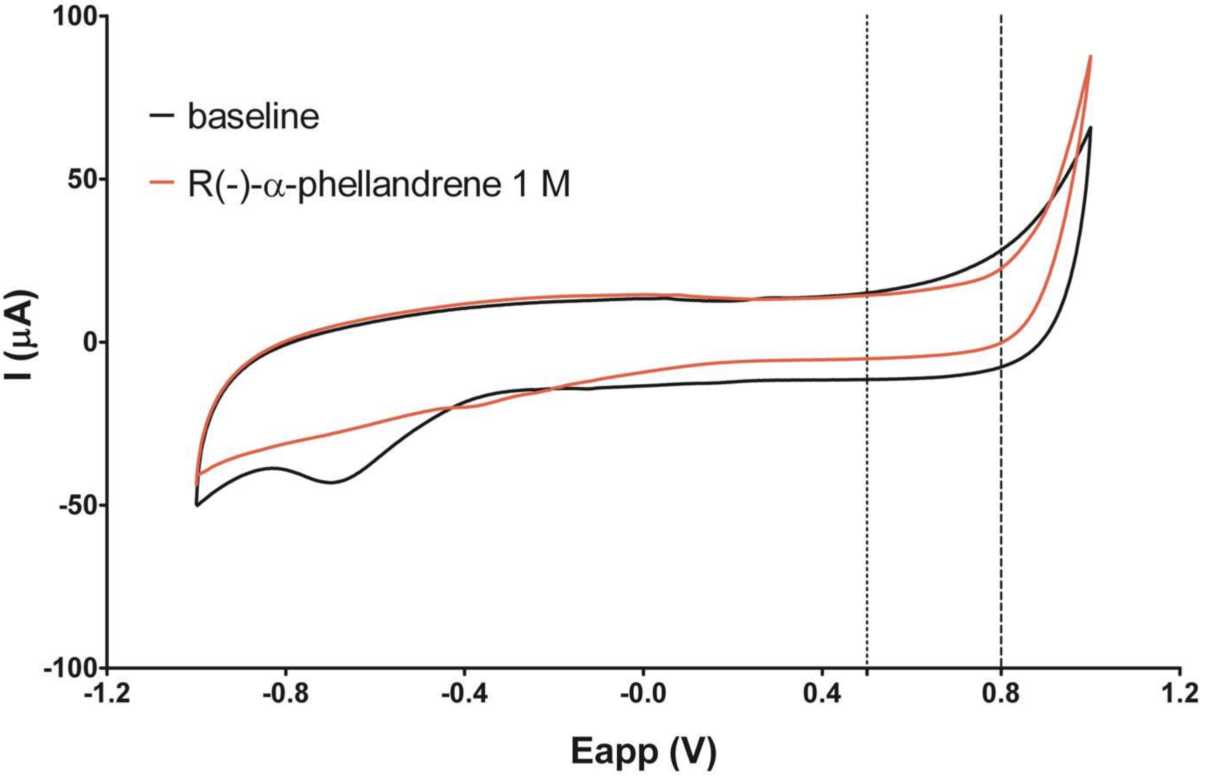

Figure S3. Cyclic voltammetry of α-phellandrene with a scanned potential range (E_{app}) comprised between - 1 V and + 1 V *vs* Ag/AgCl reference electrode, in the absence (black line) and in the presence of 1M α-phellandrene (red line).
